# Jugular foramen versus hypoglossal canal in axial CT scan

**DOI:** 10.37796/2211-8039.1393

**Published:** 2023-03-01

**Authors:** Maryam Mohammadzadeh, Reza Erfanian, Saman Rezaeian, Nasim Batavani, Behrooz Amirzargar

**Affiliations:** a Department of Radiology, Division of Neuroradiology, Tehran University of Medical Sciences, Tehran, Iran; b Otorhinolaryngology Research Center, Department of Otorhinolaryngology-Head and Neck Surgery, Tehran University of Medical Sciences, Tehran, Iran

**Keywords:** Jugular foramen, Hypoglossal canal, Computed tomography scan

## Abstract

**Background:**

Differentiating jugular foramen from hypoglossal canal in computed tomography (CT) scan is vital for correct diagnosis of posterior fossa pathologies; however, it has been shown that the ability for differentiating these skull base elements is limited. The purpose of this study was to produce a simple algorithm for differentiating the jugular foramen from the hypoglossal canal in axial CT scan on two levels (top level where bony carotid canal is evident and lower level where bony carotid canal is not evident).

**Methods:**

Data derived from axial CT scan of 250 patients (500 sides) were used for producing algorithm. At top level petro-occipital fissure utilized for recognizing occipital condyle in which hypoglossal canal is located; and, at lower level the distance between the posterior border of the anatomic element (jugular foramen or hypoglossal canal) and the tangent to the anterior bony part is used for producing algorithm.

**Results:**

The mean age of patients was 38.1 ± 19 years. The petro-occipital fissure can be used in all patients for differentiating hypoglossal canal. At lower level the distance between the anterior tangent and the posterior border of the element was significantly lower for hypoglossal canal (P value < 0.001). The distance more than 3.5 mm with sensitivity 83.8% and specificity 97.1% differentiate jugular foramen from hypoglossal canal.

**Conclusion:**

Simple algorithms based on quantitative morphologic features of the jugular foramen and hypoglossal canal can be used with high sensitivity and specificity to distinguish these elements.

## 1. Introduction

The jugular foramen is a bony canal located between the temporal bone and the occipital bone. This foramen is divided by a fibrous or bony septum into two parts, pars nervosa and pars vascularis (pars venosa). Pars nervosa is located in the anteromedial part and the inferior petrosal sinus and glossopharyngeal nerve (cranial nerve 9) pass through it. Also, pars vascularis is located in the posterolateral part and the vagus nerve (cranial nerve 10), accessory nerve (cranial nerve 11), jugular bulb and posterior meningeal artery pass through it [1,2].

The hypoglossal canal is located above the condyle of the occipital bone and anterolateral to the foramen magnum. This canal runs in a posteromedial to anterolateral direction. The hypoglossal nerve, the meningeal branch of the ascending pharyngeal artery, and an emissary vein from the basilar plexus pass through this canal [3,4].

The shape and size of the jugular foramen can be very different. Even in one person, the size and shape of this foramen may not be the same on both sides [5,6]. The hypoglossal canal is also often asymmetric and may vary in size from 3 to 10 mm [7].

The jugular foramen is closely adjacent to the hypoglossal canal. It is located below the internal acoustic meatus and superolateral to the intracranial orifice of the hypoglossal canal. Due to this close proximity and considering the differences in tumor management, surgical complications and different surgical approaches, it is important to differentiate jugular foramen pathologies from hypoglossal canal ones. This differentiation is especially difficult in large tumors [8,9].

According to experimental studies conducted among otolaryngologists and residents in this field, due to different forms of jugular foramen, it is easily possible to confuse this foramen with hypoglossal canal in axial computed tomography (CT) scan [10].

Therefore, in this study, accuracy of quantified morphologic parameters of jugular foramen and the hypoglossal canal in axial sections of CT scan was examined for the purpose of differentiation. Accordingly, a decision tree model was created using classification and regression tree (CART). CART is a form of binary recursive partitioning. In a binary decision tree, each group of cases is represented by a node, and each node can be split into two child nodes. CART model is simpler than other commonly used statistical methods and it can be applied in clinical and radiological settings to assist in decision making [11,12].

## 2. Methods

In this study, 250 patients were selected randomly. They had undergone CT scans from skull base for diagnostic purposes. Scans were performed with SIEMENS 16-slice MDCT scanner H80s very sharp FR with the following parameters: collimation of 0.625 mm; 110 kV; 100 mAs; and a matrix of 512 × 512. Axial slices of CT scans were extracted from the hospital’s picture archiving and communication system (PACS). The ethics committee of the Tehran University of Medical Sciences also approved the study protocol (IR.TUMS.VCR.REC.1397.500).

Individuals who met the inclusion criteria included the patients who underwent CT scan of the skull base with indications including otomastoiditis, primary, secondary and recurrent cholesteatoma, and the patients who were cochlear implant candidates.

In the presence of any extensive destructive process, including tumor, inflammatory, infectious, and severe traumatic lesions that resulted in the loss of a significant portion of the skull base that hinders accurate judgment regarding major anatomical landmarks, the CT scan was excluded from the study. Also, in cases of low-quality CT scans for any reason, including patient movement, artifacts or technical problems, those CT scans were excluded from the study.

**Fig. 1:**
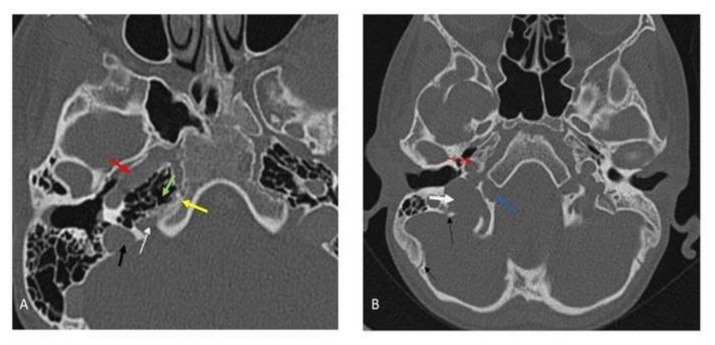
A, When the bony carotid canal is evident, the jugular foramen passes through the continuum of medial aspect of a bony segment of the carotid canal, bony part of the carotid canal is separated from occipital bone by a petro-occipital fissure. Carotid canal (Red arrow), petrous apex medial bony border of the carotid canal (Green arrow), pars nervosa of the jugular foramen (White arrow), pars venosa of the jugular foramen (Black arrow), petro-occipital fissure (Yellow arrow). B, Hypoglossal canal (Blue arrow) passing through the condyle (Bone medial to the carotid canal (Red arrow)). The jugular foramen (White arrow) passes along the medial surface of the petrous bone and in this case, the jugular foramen passes through occipital bone to connect with the posterior cranial fossa. Black arrows show a suture line between the occipital bone and temporal bone.

In this study, only parts of the jugular foramen and the hypoglossal canal that were directly connected to the intracranial space were examined. It was hypothesized that in the upper regions of the jugular foramen where the bony carotid canal is visible, all the foramina on the bone segment along the continuum of the medial part of the bony carotid canal (both temporal and occipital parts), are not hypoglossal canal and therefore they are jugular foramen or internal auditory canal. Also, the canal that passes through the occipital condyle (The bone that is adjacent the medial surface of the petrous bone and separated from bony medial border of carotid canal by petro-occipital fissure) will be the hypoglossal canal (Fig. 1). This hypothesis was evaluated in this study through investigating all CT scans.

In the lower parts of the skull, where the bony carotid canal is not visible, the indirect effect of sigmoid sinus pulse on the morphology of jugular foramen was evaluated and it was hypothesized that the sigmoid sinus pulse would cause the posterior border of the jugular foramen to recede anterior border. Sigmoid sinus pulse will create more obtuse angles between the tangents drawn to the bone which located on the anterior and posterior borders of the jugular foramen. In addition, it would create more distance between tangents drawn along the anterior part of element and posterior border of element in jugular foreman in comparison with hypoglossal canal. The mentioned angle and distance for both jugular foramen and hypoglossal canal were measured in all CT scans.

For all axial pictures in the CT scan in which bony carotid canal was not visible, there was a tangent to the anterior bony part of the element with the smallest angle to the sagittal plane drawn in each axial slices in the CT scan. Then, a vertical line was drawn from the posterior border of the element to this line and this distance was recorded in millimeters.

**Fig. 2:**
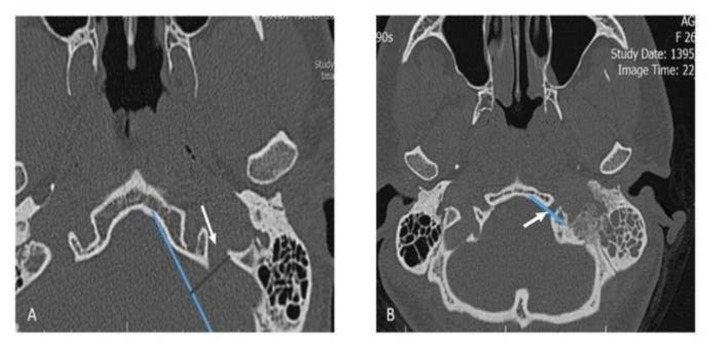
A, The distance of the posterior border of the desired element (jugular foramen, white arrow) from the tangent to the anterior bony part of the element with the lowest angle to the sagittal plane. B, The tangent (Blue line) to the bony part of the desired element (hypoglossal canal, white arrow) passes through the element, in this case, the distance is considered zero.

To challenge the mentioned hypothesis, the shortest distance for the jugular foramen and the longest distance for the hypoglossal canal on each side through all slices of each subject were recorded. If the tangent passed through the element, the distance was considered zero (Fig. 2).

**Fig. 3:**
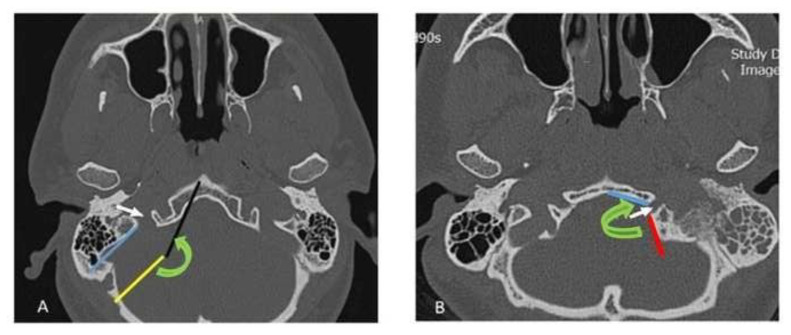
A, The angle (Green arc) between the tangent (Black arrow) drawn on the anterior part of the desired element (jugular foramen, White arrow) with the lowest angle to the sagittal plane and the tangent drawn from the posterior of the element (Blue line). To measure this angle parallel to the blue line, the yellow line intersects the black line and the angle is calculated. B, On the anterior bony part of the desired element (hypoglossal canal, White arrow), the first tangent was drawn with the lowest angle in the sagittal plane (Blue line) and the second tangent on the bony part of the posterior border of the desired element (Red line). The angle between these two lines (Green arc) was measured in degree.

For all axial pictures in the CT scan in which jugular foramen and hypoglossal canal was visible and bony carotid canal was not visible, there was a tangent to the anterior bony part of the element with the lowest angle to the sagittal plane. The second tangent was drawn to the posterior border of the element. Then, the angle between these two lines was measured. To challenge the hypothesis, the minimum angle for the jugular foramen and the maximum angle for the hypoglossal canal were selected through all axial slices in CT scan for each subject (Fig. 3). All these procedures were performed by two professional radiologists.

Data analysis was performed using Statistical Package for the Social Sciences (SPSS) software version 24. The difference between distance and angle of jugular foramen and hypoglossal canal was evaluated by paired sample t-test.

Minitab software version 19.2020.1 was used to create the differential algorithm base on CART. Jugular foramen or hypoglossal canal was considered as response. The input data were age, sex, side, the angle between the anterior and posterior tangents, and distance of element to anterior tangent. K-fold cross validation was considered as the validation method and K was considered as 10. The minimum number for splitting at each node was considered to be 10. An optimal model derived by CART algorithm extracted for distinguishing jugular foramen from the hypoglossal canal.

For calculating sample size, we used the general rule for the clinical prediction model, for each parameter we needed 10 to 50 samples for building a reliable model [13]. In our study, we included 5 parameters (sex, age, side, angle, and distance). Therefore, we used 250 cases for building a reliable and powerful model.

## 3. Results

Totally, 250 patients were evaluated in the study. The mean age was 38.1 ± 19 years (between 1 and 90 years). Among them, 39.6% of patients were women and 60.4% were men.

In all analyzed cases in which the bony carotid canal was evident, the jugular foramen passed directly adjacent to continuum of medial border of carotid canal toward occipital bone and hypoglossal canal passed always through bone which was located medial to bony carotid canal with intervening soft tissue spacer.

In the analysis of the angle on the right, the angle between the anterior tangent with the most medial angle and the tangent with the posterior border of the hypoglossal canal was 167.1 ± 27.0°. This angle for jugular foramen was 219.8 ± 21.0°. This difference was statistically significant (P value < 0.001).

On the left, the angle between the anterior tangent and the tangent to the posterior border of the hypoglossal canal had a mean of 172.0 ± 28.5. For the jugular foramen, this angle was 220.9 ± 19.4 and the difference was statistically significant (P value < 0.001) (Table 1).

The hypoglossal angle in men and women was 169 ± 26.79° and 163.83 ± 27.3° on the right and 173.49 ± 29.35° and 169.70 ± 27.14° on the left, respectively.

The jugular angle in men and women was about 219.99 ± 21.45° and 219.92 ± 20.02° on the right and 223.35 ± 17.89° and 216.69 ± 21.32° on the left, respectively. Hypoglossal and jugular angles did not show a statistically significant difference between men and women.

On the right, the distance between the posterior border of the hypoglossal canal to the anterior tangent was 0.28 ± 1.0 mm and the distance between the posterior border of the jugular foramen and the anterior tangent was 9.83 ± 4.3 mm. T-test indicated that there was a statistically significant difference (P value < 0.001).

On the left, the distance between the posterior border of the hypoglossal canal to the anterior tangent was 0.19 ± 0.8 mm and the distance between the posterior border of the jugular foramen and the anterior tangent was 9.90 ± 4.0 mm which showed a significant difference (P value < 0.001).

The difference between these angles in men and women was not significant. Tables 2 and 3 summarize these findings.

**Fig. 4:**
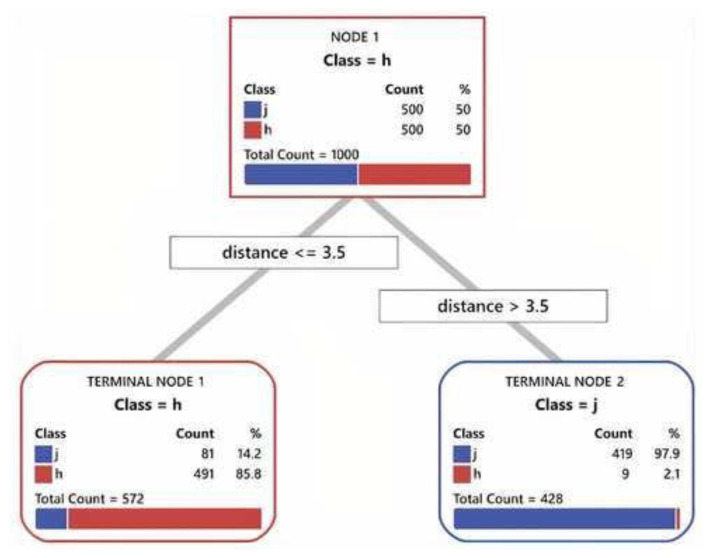
CART diagram for the differentiation of jugular foramen from the hypoglossal canal when the bony carotid canal is not evident. The calculated distance between the posterior border of the anatomic element and the tangent to the anterior bony part for differentiation was 3.5 mm.

CART diagrams for the elements are drawn in Fig. 4. The relative importance of differentiating the two parameters of distance and angle for differentiating the jugular foramen from the hypoglossal canal was 100 and 65, respectively. The area under the curve for the training and test was 0.91 and 0.89, respectively. Its sensitivity for training and test for jugular foramen was 83.8% and 83.6% and specificity for training and test for jugular foramen was 98.2% and 97.6%. The confidence interval was 0.87–0.91 in the test model.

## 4. Discussion

In the present study, a cut off for differentiating jugular foramen from hypoglossal canal was introduced in single axial slice of CT scan without the need to evaluate other adjacent CT scan slices. In this study, an attempt was made to benefit from the morphologic impact of sigmoid sinus pulse on pars venosa of jugular foramen for differentiation. In axial slices in which the pars nervosa part of jugular foramen is evident and there is no sigmoid sinus pulse impact, medial border of bony carotid canal continuum toward occipital bone was used as a clue for finding jugular foramen and differentiating it from hypoglossal canal which passed through bony condyle and was separated from bony carotid canal by intervening soft tissue.

The right jugular foramen is larger than the left in 68% of people. In the inferomedial region, jugular foramen separates from the hypoglossal canal by the jugular tubercle. The hypoglossal canal is located inside the occipital bone and inferior to the jugular foramen and is very close to the jugular foramen. This canal passes through the occipital condyle laterally and anteriorly. The hypoglossal canal is measured as 7.8 by 5 mm and is directed inferiorly, laterally and anteriorly [14–16].

The pathological lesions involving the jugular foramen and the hypoglossal canal are different. Likewise, different surgical approaches are used to access these canals [17–19]. Accordingly, the differentiation of these two elements is of paramount importance in imaging studies [20–21]. Although getting access to the hypoglossal canal may be similar as for the jugular foramen, sometimes utilizing a more conservative approach for benign lesions of jugular foremen is appropriate as it can be associated with the vagus nerve injury which may be associated with morbidity and mortality especially in elder people [9]. In addition, access to the hypoglossal canal by craniotomy approaches needs jugular tubercle drilling which best achieved by the far lateral approach [22]. The development of endoscopic approaches to the hypoglossal canal obviates the need for foramen jugular elements dissections which paramount differentiation of the hypoglossal canal from the jugular foramen in benign pathologies [23]. The proposed model can be used for distinguishing these two elements automatically.

One of the strategies for finding hypoglossal canals in axial CT scan is to find the atlantooccipital joint and the condyle of occipital bone, and then find the bony defect inside the condyle [15]. However, jugular foramen may pass through occipital bone as Fig. 1 manifests, thereby making the rule ineffective. The main problem for differentiating jugular foramen from hypoglossal canal is various and complicated compartmentation of jugular foramen which is different in lower and upper segments [24]. In this study we divide jugular foramen based on vertical levels into two levels, at top level the carotid canal with bony borders are evident and jugular foramen has two distinct compartments of pars venosa and pars nervosa; in addition, petro-occipital fissure is evident. At this top level the occipital condyle is separated from petrous bone by petro-occipital fissure clearly and hypoglossal canal pass through this thick bone and jugular foramen territory is located on medial surface of petrous bone. Because the sigmoid sinus is far from the pars vasularis and there is pars nervosa the indirect effect of sigmoid sinus pulse on morphology of jugular foramen can’t be utilized for differentiation at top level. At lower axial slices where the bony carotid canal is not evident and pars vascularis and pars nervosa were unified we can use pulse effect of sigmoid sinus which push posterior part of jugular foramen anteriorly.

“Eagle beak sign” is a reliable landmark for differentiating the hypoglossal canal from the jugular foramen [25] but this sign is only apparent in coronal slices and by our approach, we obviate the need for multipolar reconstructions which is usually available by PACS. The presented algorithm also obviated the need for scrolling up and down the radiologic images for discrimination of these basic elements and provided better situation for focusing on the main problem especially for complicated skull base cases and during surgery.

After reviewing the literature, to the best of the authors’ knowledge, there was no study providing the criteria for differentiating the jugular foramen from the hypoglossal canal in axial CT scan. According to our information, our study is the first study that specifically investigated the methods to differentiate the two in CT scan slices. In this study, the CART model was used to differentiate outcomes. CART model is a powerful technique for presenting data splitting algorithms that enables to produce a binary decision tree and classification system, based on the variables that are most likely to differentiate between outputs [26].

The use of the CART model in the analysis of clinical studies is increasing. This model has been used in several studies in various clinical fields [26–28]. Moreover, some radiological studies have used this model to differentiate the findings [29,30]. The advantage of this model is merging all available variables for building the most accurate classification tree for decision making. This study is the first to use the CART model to accurately differentiate the jugular and hypoglossal elements in CT scan imaging of the skull base. This algorithm not only can help radiologists and surgeons but also can be applied in advanced artificial intelligence for differentiating these basic and fundamental elements.

Using the cut-off limit determined in this study and the tree diagrams presented, helps physicians to differentiate the jugular foramen from the hypoglossal canal with high sensitivity and specificity.

Although both distances and angles allow the jugular foramen to be differentiated from the hypoglossal canal with high sensitivity and specificity, the distance between the two is more powerful than the angle.

In this study, normal-scale CT scans of skull base were examined and in case of any lesion and disruption, the relevant CT scan was not included in the study. Applying the results of this study which conducted on normal-scale CT scans can be challenging in cases where the morphology of the skull base foramina has changed as a result of any pathological process. Considering that the ultimate goal of this study was to help differentiate between the jugular foramen and the hypoglossal canal in pathological situations and to identify the tumors of this area, further studies and validation of diagnostic tests should be conducted in pathological cases.

## 5. Conclusions

Simple algorithms based on quantitative morphologic features of the jugular foramen and hypoglossal canal can be used with high sensitivity and specificity to distinguish these elements based on CART model. This finding can help radiologists and surgeons to discriminate these basic and important elements in axial slices of CT scan without the need to scroll up and down the CT scan slices.

## Figures and Tables

**Table 1:** Angle (in degrees) between the anterior tangent with the most acute angle and the tangent to the posterior border of the element.

	Minimum	Maximum	Mean	Std. deviation
Maximum Right hypoglossal angle	104	260	167	27.06
Maximum Left hypoglossal angle	120	280	172	28.51
Minimum Right jugular angle	153	260	219	20.88
Minimum Left jugular angle	140	260	220	19.44

**Table 2:** Distance (in mm) between the posterior border of the jugular foramen and the anterior tangent in sex groups.

Side	Sex	Mean	Std. deviation	p-value
Right (9.83)	Male	9.92	4.54	0.845
	Female	9.69	3.87	
Left (9.90)	Male	10.29	4.10	0.071
	Female	9.25	3.64	

**Table 3:** Distance (in mm) between the posterior border of the hypoglossal canal and the anterior tangent in sex groups.

Side	Sex	Mean	Std. deviation	p-value
Right (0.28)	Male	0.36	1.19	0.293
	Female	0.16	0.55	
Left (0.19)	Male	0.25	0.97	0.332
	Female	0.10	0.46	
